# Attributes of intestinal microbiota composition and their correlation with clinical primary non-response to anti-TNF-α agents in inflammatory bowel disease patients

**DOI:** 10.17305/bjbms.2021.6436

**Published:** 2021-11-10

**Authors:** Hanan Alatawi, Mahmoud Mosli, Omar I. Saadah, Vito Annese, Rashad Al-Hindi, Marfat Alatawy, Hadba Al-Amrah, Dikhnah Alshehri, Ahmad Bahieldin, Sherif Edris

**Affiliations:** 1 Department of Biological Sciences, Faculty of Science, King Abdulaziz University, Jeddah, Saudi Arabia; 2 Department of Biological Sciences, University College of Haqel, University of Tabuk, Tabuk, Saudi Arabia; 3 Department of Internal Medicine, Faculty of Medicine, King Abdulaziz University, Jeddah, Saudi Arabia; 4 Department of Pediatrics, Faculty of Medicine, King Abdulaziz University, Jeddah, Saudi Arabia; 5 Inflammatory Bowel Disease Research Group, King Abduklaziz University, Jeddah, Saudi Arabia; 6 Department of Internal Medicine, Fakeeh University Hospital, Dubai, United Arab Emirates; 7 Department of Genetics, Ain Shams University, Cairo, Egypt; 8 Princess Al Jawhara Albrahim Centre of Excellence in Research of Hereditary Disorders (PACER-HD), King Abdulaziz University, Jeddah, Saudi Arabia

**Keywords:** Inflammatory bowel disease, non-responders, microbiota

## Abstract

The largest microbial aggregation in the human body exists in the gastrointestinal tract. The microbiota in the host gastrointestinal tract comprises a diverse ecosystem, and the intestinal microbiota plays a key role in maintaining gut homeostasis. This study aims to examine whether the gut microbiota influence unresponsiveness to anti-TNF-α treatments in primary non-responder patients and consequently identify the responsible microbes as biomarkers of unresponsiveness. Stool samples were collected from a cohort of patients with an established diagnosis of IBD, either ulcerative colitis or Crohn’s disease, following completion of the induction phase of anti-TNF therapy. 16S rRNA sequencing analysis was used to examine the pattern of microbiota communities in fecal samples. The quality and quantity of fecal microbiota were compared in responder and primary non-responder IBD patients following anti-TNF-α therapy. As per our hypothesis, a difference in gut microbiome composition between the two patient subgroups was observed. A decreased abundance of short-chain fatty acid (SCFA)-producing bacteria, including *Anaerostipes, Coprococcus, Lachnospira, Roseburia, and Ruminococcus*, was detected in non-responsive patients, which was the hallmark of dysbiosis. Biomarkers of dysbiosis that was identified as predictors of clinical non-response included *Klebsiella, Eubacteriaceae, RF32, Bifidobacterium animalis*, and *Muribaculaceae* previously known as *S24-7*. Signature biomarkers showed dramatic alteration in the composition of gut microbiota in patients who demonstrated primary non-response to anti-TNF-α agents. Dysbiosis, with features including a dropped biodiversity, augmentation in opportunistic pathogenic microbiota, and a lack of SCFA-producing bacteria, is a prominent feature of the microbiome of primary non-responders to anti-TNF-α therapy.

## INTRODUCTION

Ulcerative colitis (UC) and Crohn’s disease (CD) are the primary subtypes of inflammatory bowel disease (IBD), found in individuals with genetic susceptibility subject to particular environmental conditions [1]. IBD is a chronic and relapsing inflammatory condition of the gastrointestinal tract that occurs following immune system dysregulation [2]. Common clinical symptoms of IBD are abdominal pain, diarrhea, weight loss, and bloody stools [3,4].

One hundred trillion different microorganisms live in the human gut, including bacteria, viruses, fungi, and protozoa [5]. According to various molecular studies in a number of different cultures with various genetic profiles, more than 1000 species of microbiota colonize the gastrointestinal ecosystem, including *Firmicutes*, *Bacteroidetes*, *Proteobacteria*, and *Actinobacteria* [2,6].

The intestinal microbiota performs a vital role in the degradation of indigestible carbohydrates to produce short-chain fatty acids (SCFAs) and is important for vitamins synthesis (Vitamin K, Vitamin B12, and folic acid), amino acids synthesis, and regulation of fat metabolism. All these processes are necessary for the preservation of intestinal barrier functions [7]. The phyla *Firmicutes* and *Bacteroidetes* use the indigestible carbohydrates to produce SCFAs with the cooperation of bacteria specialized in the process of oligosaccharide fermentation, such as *Bifidobacteria* [8]. SFCAs – acetate, propionate, and butyrate – are the fundamental and essential end products of carbohydrate fermentation in the colon. Microbiota present in colonic epithelial cells consume butyrate to produce energy, while acetate and propionate remain in the intestinal cell [9].

Dysfunction in the composition and abundance of gut microbiota is associated with IBD severity. A significant decrease in the diversity of commensal bacteria, such as *Bacteroides, Lactobacillus, Ruminococcus, Faecalibacterium*, and *Bifidobacterium*, has also been observed in IBD patients. Low diversity of *Roseburia* spp. is associated with a high risk of IBD pathology [10].

A precise definition of primary nonresponse in IBD patients has not been established, nevertheless, an accepted definition with respect to the employment of anti-TNF therapy is a failure to fulfill clinical remission following the induction of a remedy period with documentation of adequate drug levels [11]. The role of the intestinal microbial composition in primary non-responders is not well understood. Several studies have shown no significant dissimilarity in the gut microbiome profile pre- and post-treatment with anti-TNF antagonists [12].

This study aims to determine whether the composition of gut microbiota has an effect on initial unresponsiveness to anti-TNF-α treatments in IBD patients. Accordingly, this study sets out to identify responsible microbiota and posits the utilization of the microbiota pattern as a biomarker to indicate unresponsiveness.

## MATERIALS AND METHODS

Twenty Saudi Arabian IBD patients were recruited for this study from the outpatient Gastroenterology Department at King Abdulaziz University Hospital, in Jeddah, Kingdom of Saudi Arabia. The inclusion criteria were male or female patients that were 20–45 years old with a confirmed diagnosis of IBD, established through clinical, endoscopic, and histological criteria. Case record forms were obtained from patients, which included demographic and clinical data regarding gender, age, marital status, family history, diagnosis reports, environmental exposures (e.g., smoking), body mass index, and various laboratory parameters. The study was designed for patients treated with anti-TNF-α antagonists only (infliximab and adalimumab). The study mainly compared IBD patients that demonstrated no response (primary non-responders) to anti-TNF agents (n = 10) and patients that were judged to be responders to anti-TNF-α antagonists (n = 10). Primary non-response was defined as failure to demonstrate clinical remission following completion of the induction period anti-TNF therapy, that is, adalimumab or infliximab, without TDM. Similarly, response was defined as demonstration of clinical response or clinical remission at the end of induction.

The exclusion criteria were patients with contraindications to anti-TNF-α antagonists, including active tuberculosis, sepsis, or other severe opportunistic infections. Participants that were treated with medication, such as antibiotics, corticosteroids, mesalamine, and immunosuppressants, within 3 months before fecal collection were also excluded, as were pregnant or breastfeeding women and those that failed to submit stool samples as described at each phase of the study.

### Sample collection and genomic DNA extraction

The fecal samples were collected, following completion of the induction phase of anti-TNF therapy, using the iSWAB-Microbiome Collection Kit and stored frozen at −20°C within 24 hours of collection. Genomic DNA of the gut microbes was extracted from the 200 μL fecal sample using PureLink^™^ Microbiome DNA Purification Kit (Invitrogen by Thermo Fisher Scientific, USA), following the instructions set out in the International Human Microbiome Standards project:

http://www.human-microbiome.org/

### Polymerase chain reaction (PCR) DNA amplification and library preparation

Isolated genomic DNA of 20 stool samples was amplified for the target V3-V4 hypervariable regions of 16S rRNA, an intestinal bacteria DNA, using the universal primer set, Primer 341 Forward (5’-CCTACGGGNGGCWGCAG-3’) and Primer 806 Reverse (5-GACTACHVGGGTATCTAATCC-3’). The total PCR comprised 25 μL, as follows: 7.5 μL H2O, 12.5 μL DreamTaq PCR Master Mix, 3 μL DNA genome, and 1 μL forward/reverse primers. The thermal cycling condition of the PCR was carried out using an initial denaturation step at 95°C for 5 minutes followed by 40 cycles at 95°C for 30 seconds (denaturation), 56°C for 30 seconds (annealing), 72°C for 30 seconds (extension), and a final extension step at 72°C for 10 minutes, and a 4°C hold. A 5 μL of 5X DNA Loading Buffer Blue was added to the PCR product.

### 16S bioinformatics sequence analysis

Raw reads were refined using the Illumina MiSeq platform [13]. Paired end reads were generated using FLASH (v1.2.11) [14]. The reads sequenced with tags were clustered to operational taxonomic units (OTUs) at 97% sequence similarity using the USEARCH software (v7.0.1090) [15]. OTU representative sequences were classified according to the databases of 16S rDNA, with the Ribosomal Database Project Classifier (v.2.2) and Greengenes database [16], using 0.6 CI as a cutoff. A representative OTU phylogenetic tree was generated using the QIIME software (v1.80) (https://qiime2.org). A pre-filtering of selected OTUs was done according to the richness and evenness; at the initial analysis, richness and evenness were checked in all samples and OTUs that did not represent the richness and evenness in all samples were excluded from the study. The microbial alpha diversity indices were computed with the Mothur software (v1.31.2), and the microbiota beta diversity distance was generated by QIIME (v1.80). All unclassified bacteria features were discarded.

### Ethical statement

This study was approved by the Research Committee, Unit of Biomedical Ethics, Faculty of Medicine at King Abdulaziz University, Jeddah, Saudi Arabia (Ref. No. 372-19). Informed consent was obtained from all patients.

### Statistical analysis

An unpaired parametric *t-*test was incorporated using the Prism 9.0 software (GraphPad Software, Inc., San Diego, CA) and R (v3.1.1) to compare quantitative variables between groups and to identify taxa that had statistically significant differences. The resulting *P* value was modified using the Benjamini-Hochberg false discovery rate correction formula (function “p.adjust” in the stats package of R, v3.1.1). PERMANOVA was used to test significance among values. The linear discriminant analysis effect size (LEfSe) method was incorporated to reveal metagenomic biomarkers. The Pearson correlation coefficient was performed with a two-tailed test and a 95% CI in GraphPad Prism 9.0. Any differences between groups considered to be significant had *p* < 0.05.

### Availability of data and material

The datasets (raw data), which supported the findings of the study, were deposited in the National Centre for Biotechnology Information (NCBI) with the unique BioProject ID: PRJNA673078 (https://dataview.ncbi.nlm.nih.gov/object/PRJNA673078).

## RESULTS

### Baseline characteristics

The study cohort included 20 patients with IBD (14 CD and 6 UC). The median age was 28 years (range, 20–45 years). Females constituted 65% (n = 13) of the cohort. The anti-TNF alpha treatment comprised infliximab in 11 patients (65%) and adalimumab in 9 patients (45%). Details of the study group are shown in Table 1.

**TABLE 1:**
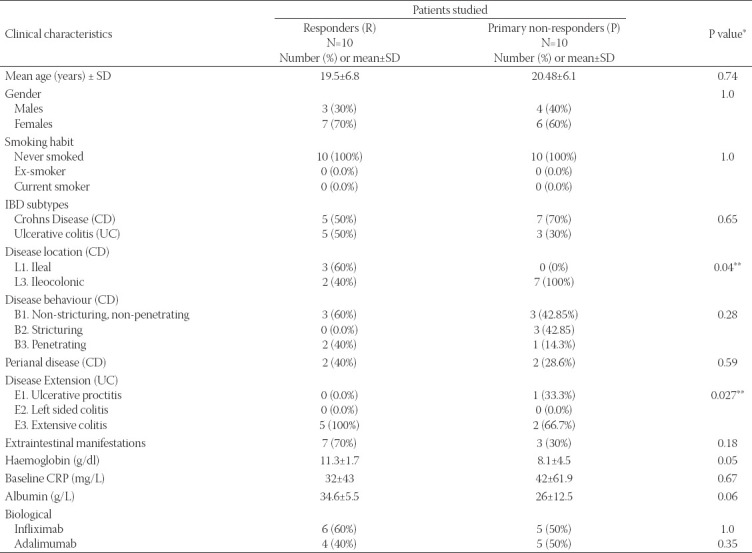
Clinical and demographic characteristics of IBD patient responders and non-responders to anti-TNF-α therapy.

### Comparison of relative abundance of microbial community structure

**FIGURE 1:**
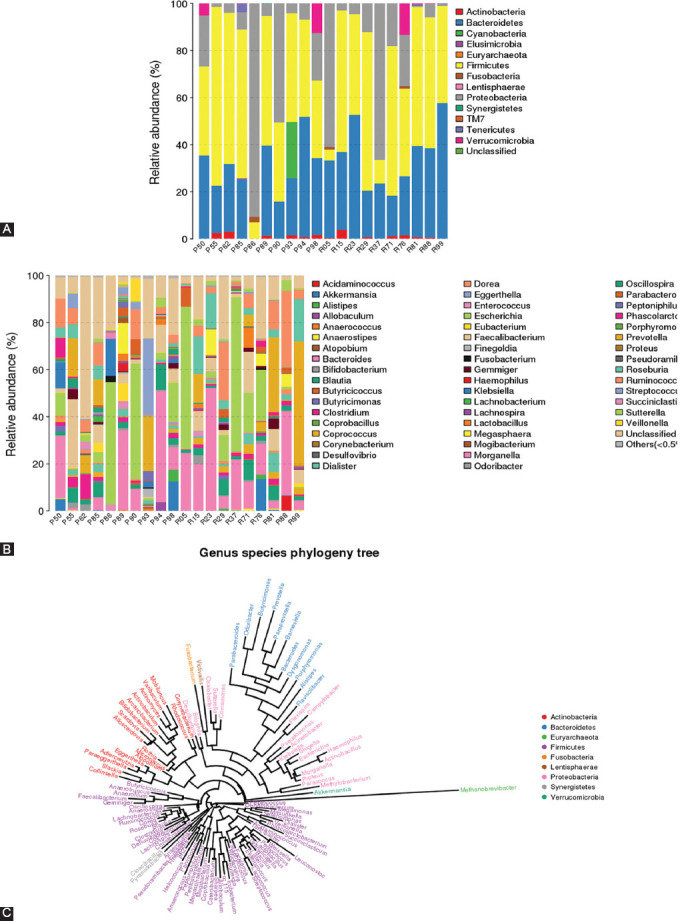
The taxonomic composition distribution of relative abundance (A) at the phylum level, (B) the genus level among the samples of both groups; (P) refers to primary non-responders and (R) to responders. (C) is a phylogenetic tree showing the evolutionary distance in biological taxa.

**FIGURE S1:**
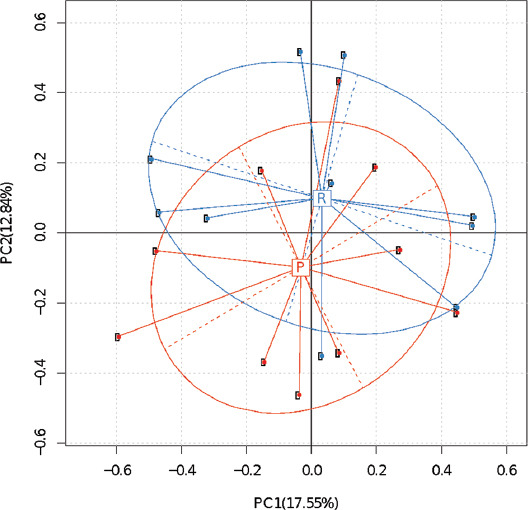
PCA based on OTU abundance. The X-axis shows 1st principal component and Y-axis shows 2nd principal component. The percentages in parentheses represent contributions of principal components to differences among samples. A colored dot represents each sample: primary non-responders (P) = blue, and responders (R) = red.

The OTUs median number was statistically estimated to be 146 for primary non-responder patients (P) and 158 for responders (R). The expressed sequence tags were clustered into OTU with a 97% threshold Figure S1. The taxonomic composition distribution was used to identify the intestinal microbiota of IBD patients at phylum and genus levels [Figure 1A and B]. The largest number at the genera level was the *Firmicutes* phylum (57 genera), followed by *Proteobacteria* (18 genera), then *Actinobacteria* (18 genera), and then *Bacteroidetes* (11 genera) [Figure 1C].

**FIGURE 2:**
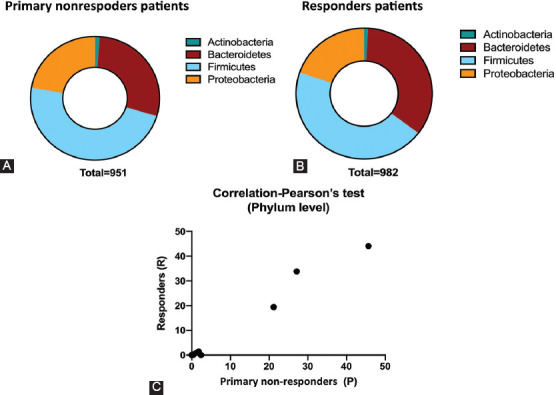
Predominant phyla: comparison of the relative abundance at the phyla level in faecal samples (A) primary non-responders, and (B) responders. (C) is a Pearson correlation coefficient graph showing r = 0.9900, 95% confidence interval = 0.9677 to 0.9969, and R squared = 0.9801.

**FIGURE 3:**
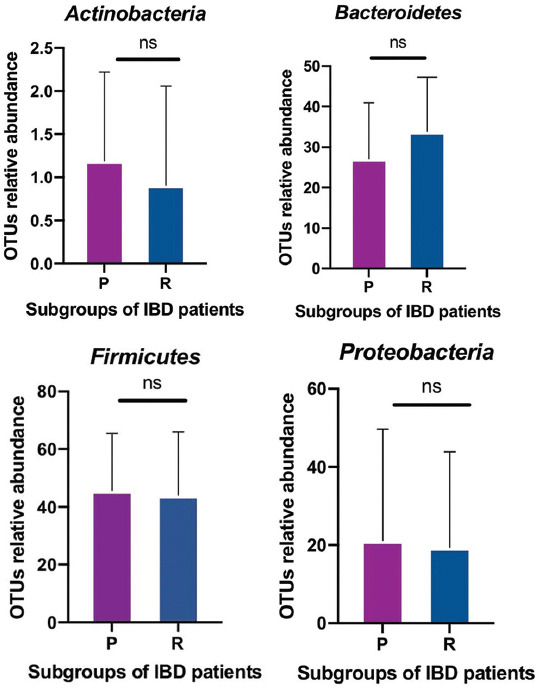
Independent samples t-test statistically showing phyla of the gut microbiota. All P values were non-significant for responders (R=blue) and primary non-responders (P=purple) to anti-TNF-α therapies.

At the phylum level, the fecal microbiota of primary non-responders were characterized by the increased relative abundance of *Proteobacteria* and *Actinobacteria* [Figure 2A] compared to responders [Figure 2B]. Interestingly, the populations of *Firmicutes* did not manifest obvious changes between the two primary subgroups of IBD patients (UC and CD) [Figure 2B]. The ratio of Bacteroidetes was lower in primary non-responders compared with responders. The Pearson correlation coefficient showed a strong positive correlation in gut microbiota composition between responder and non-responder IBD patients [Figure 2C]. Furthermore, an independent t-test showed no statistically significant difference between the relative abundance of predominant phyla of gut microbiota in response to anti-TNF-α treatment between the two IBD patient groups [Figures 3 and S1].

**FIGURE S2:**
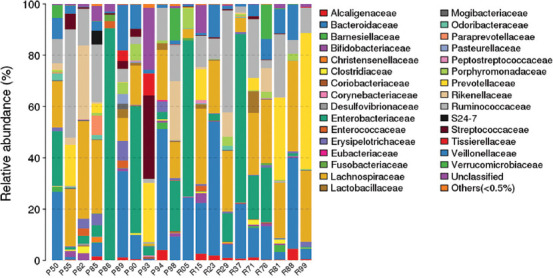
The taxonomic composition distribution in family-level across 20 samples of responders and non-responders to anti-TNF-α treatments.

At the family level [Figure S2], the relative abundance of the *Clostridiaceae* family was stable in the two subgroups of patients. However, there was a remarkable increase in several families, including *Corynebacteriaceae*, *Eubacteriaceae*, *Desulfovibrionaceae*, *Lachnospiraceae*, *Streptococcaceae*, and *Muribaculaceae* – previously known as *S24-7* – in non-responders. Furthermore, the diversity of *Lactobacillaceae*, *Ruminococcaceae*, *Alcaligenaceae*, *Mogibacteriaceae*, *Peptostreptococcaceae*, *Veillonellaceae*, *Prevotellaceae*, and *Bacteroidaceae* decreased in non-responders in comparison with responders.

**FIGURE 4:**
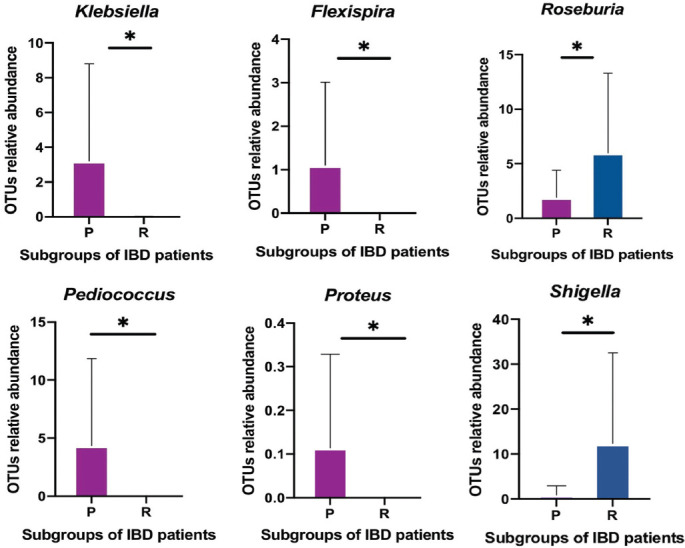
Independent samples t-test showing responders (R=blue) and primary non-responders (P=purple) with statistically significant P values in Pediococcus (*P< 0.034983), Proteus (*P< 0.040331), Shigella (*P< 0.010478), Roseburia (*P< 0.04166), Flexispira (*P< 0.034983), and Klebsiella (*P< 0.016427)

**FIGURE S3:**
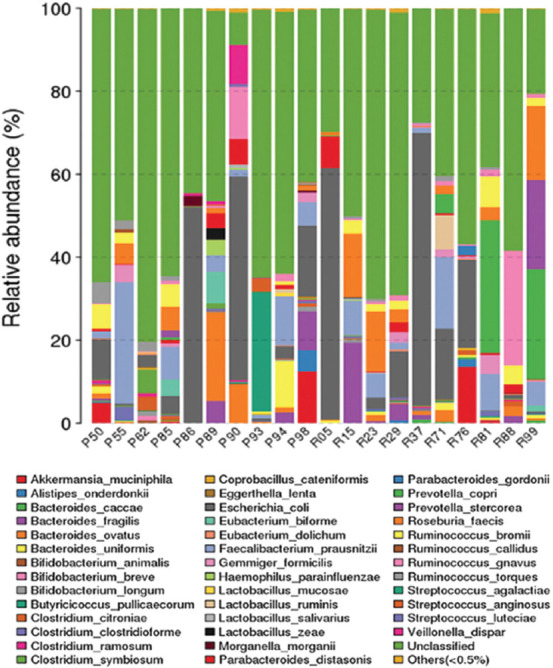
The taxonomic composition distribution in species-level across 20 samples of responders and non-responders to anti-TNF-α treatments.

At the genus level [Figure 1B] and species level [Figure S3], a rise and fall in the relative abundance of intestinal genera and species appeared to directly affect patient ability to respond to the drug. Several SCFA-producing bacteria were omitted in unresponsive patients (primary non-responders). These included *Anaerostipes, Coprococcus, Lachnospira, Roseburia*, and *Ruminococcus*. By contrast, some SCFA-producing bacteria showed an increase in relative abundance in primary non-responders, such as *Blautia, Faecalibacterium, Lachnobacterium, Odoribacter, Oscillospira*, and *Pseudoramibacter eubacterium*. An increased diversity of several pathogenetic gut microbiota was observed in primary non-responders, including *Enterococcus, Fusobacterium* (adherent-invasive bacteria), *Clostridium, Streptococcus, Staphylococcus, Actinomyces, Corynebacterium, Rothia, Rhodococcus, Atopobium, Listeria, Peptostreptococcus, Clostridium, Campylobacter, Flexispira, Morganella, Actinobacillus, Selenomonas, Haemophilus, Acinetobacter*, and *Klebsiella*. Furthermore, a great abundance of opportunistic pathogens such as *Muribaculaceae, Proteus, Odoribacter, Alistipes, Veillonella*, and *Pseudomonas* was recorded in fecal samples of primary non-responders. An independent *t*-test showed a statistically significant association (*p* < 0.05) for six genera and seven species [Figure 4]. These results suggest that primary non-responders to anti-TNF-α treatment possess a type of unstable gut microbiota structure.

### Alpha and beta diversity

**FIGURE 5:**
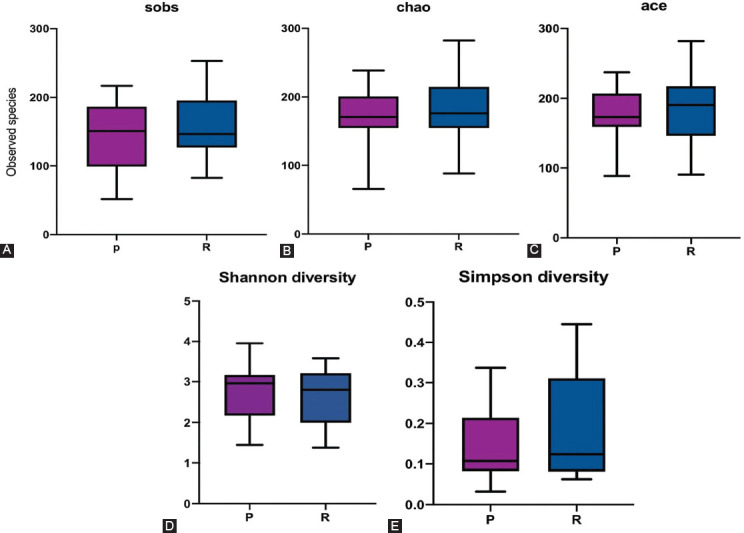
Plot of microbial alpha diversity in patients responding to anti-TNF-α treatment, calculated using observed species (A) [S_obs_], (B) Chao, (C) ACE, (D) Shannon, and (E) Simpson indices in different faecal samples. Alpha diversity was non-significant. Primary non-responders (P) = purple, and responders (R) = blue.

**FIGURE S4:**
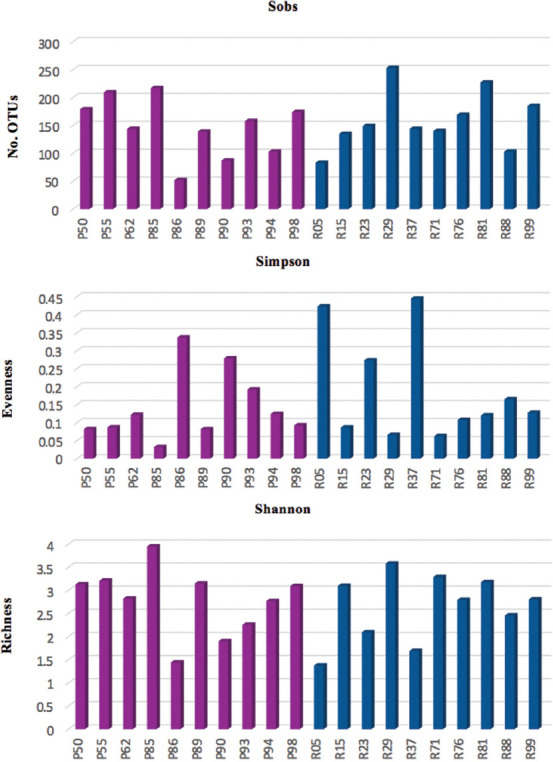
Alpha-diversity was calculated using the observed number of operational taxonomic units [OTUs], Shannon index, and Simpson index among faecal samples in primary non-responders (P) (purple) and responders (R) (blue) to anti-TNF-α treatment.

The alpha diversity was measured in observed species (S_obs_) (*p* = 0.79118), using the Chao1 estimator (*p* = 0.63053), ACE (*p* = 0.57874), Shannon diversity (*p* = 0.79594), and Simpson diversity (*p* = 0.63053) indices [Figure S4 and Table S1]. In general, in terms of alpha diversity, there was no significant difference in relative abundance between responders and primary non-responders [Figure 5 and Table S2].

**FIGURE 6:**
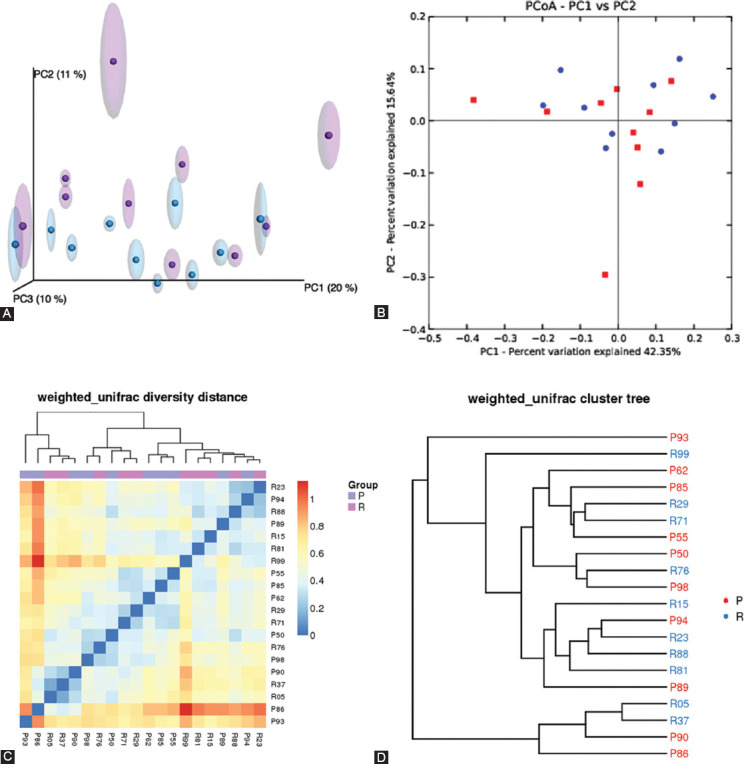
(A) Principal coordinate analysis (PCoA). (B) 2D-weighted UniFrac profile of β-diversity for microbial diversity among stool samples. (C) Weighted-UniFrac heat map. (D) Phylogenetic tree indicating presence of clear clustering in microbial structure.

**FIGURE S5:**
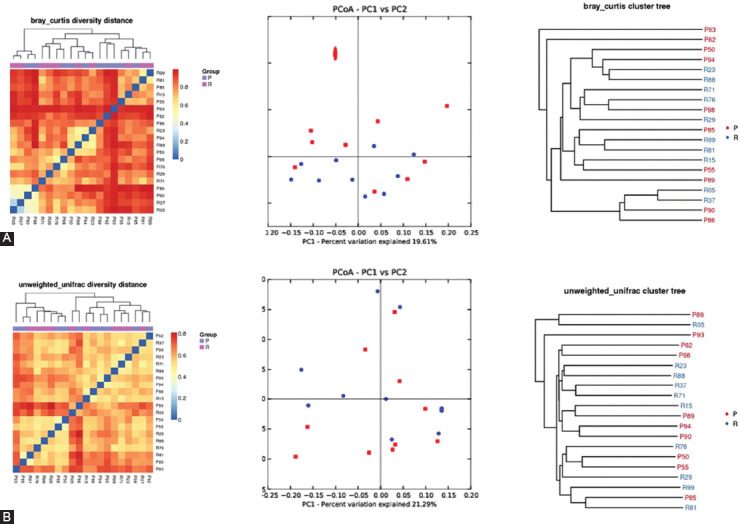
(A) Beta diversity and Bray-Curtis distance; (B) Unweighted UniFrac diversity distance. Shows the dissimilarity/similarity between the two groups, responders (R) and primary non-responders (P).

The beta diversity analysis was estimated using the Bray–Curtis dissimilarity, for unweighted and weighted UniFrac distances Figure S5. These tools showed that the composition of intestinal microbiota of primary non-responders overall shifted toward dysbiosis compared with responders. Principal coordinate analysis and 2D-weighted UniFrac showed a significant overlap between the gut microbiota structure of responders and primary non-responders [Figure 6A and B]. The weighted UniFrac heat map was used to determine the intestinal microbiota profile distances in IBD patients, between responders and non-responders. The microbial community composition of primary non-responders showed the proportion of change (disorder) compared with responders [Figure 6C]. The weighted UniFrac phylogenetic tree indicated that the community shared a lot of evolutionary history at the phylum level. However, at the genus and species level, the microbial community tends to diverge. Overall, half of the phylogenetic tree of unresponsive individuals were unique to responders, with a distance of 0.5 (fraction) [Figure 6D].

### LEfSe analysis

**FIGURE 7:**
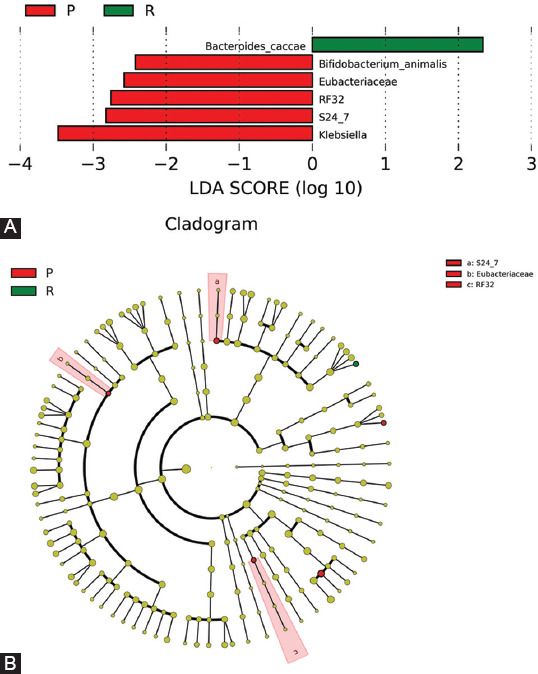
LEfSE shows (A) Negative LDA scores were enriched in primary non-responders with five taxa. Positive LDA were enriched in responders with one taxon; red (P) indicates primary non-responders and green (R) indicates responders. (B) Cladogram tree of biomarkers.

The LEfSe analysis suggests that *Klebsiella* sp. and *Bifidobacterium animalis* robustly correlated with primary non-response [Figure 7A]. Furthermore, *Eubacteriaceae*, *Streptococcaceae* (*RF32*), and *Muribaculaceae* (*S24-7*) families are characterized by non-responsiveness to anti-TNF-α agents [Figure7A and B]. Response to anti-TNF-α agents was distinguished by the presence of *Bacteroides caccae*, hence, the *Bacteroidetes* phylum can be considered as a biomarker for response.

## DISCUSSION

This study examined the microbiota pattern of IBD patients that did not respond to anti-TNF-α therapies compared to those of demonstrated response [4,17]. The gut microbiota were compared to assess the nature of the dysbiosis and identify the microbes that could potentially be responsible for dysbiosis, and consequently to a lack of response. The present study depended on next-generation sequencing technologies, especially for the 16S rRNA gene sequencing, which permits describing the gut microbiota architecture. The 16S rRNA gene sequencing is a taxonomic genomic marker that is specialized for examination of a particular part of the microbiota, the bacteria, and archaea, regardless of other microorganisms, such as virobiota, mycobiota, and eukaryota [18]. The overall vision of the study is to identify microbiota structures that could be used as a prognostic indicator for clinical non-response to anti-TNF-α therapy in Saudi Arabian patients with IBD. According to the previous literature, it has been shown that high concentrations of *Klebsiella, S24-7*, Eubacteriaceae, and *Bifidobacterium animalis* are associated with clinical nonresponse to anti-TNF-α treatment, while low concentrations of *B. caccae* can be used as a biomarker for response. Patients that demonstrated nonresponse to treatment had a characteristic reduction of SCFA-producing bacteria or a shift toward an inflammation-producing bacterium [19]. Furthermore, a greater number of opportunistic pathogens such as *Muribaculaceae, Proteus, Odoribacter, Alistipes, Veillonella*, and *Pseudomonas* were recorded in fecal samples of primary non-responders.

In this study, we did not observe a great dissimilarity in gut microbiota composition between responders and non-responders. Only moderate differences were found, with limited statistical significance. Of particular note, the majority of the findings were consistent with some previous studies on the gut microbiome in the context of different diseases, such as spondyloarthritis [12,20]. An increased abundance of *Proteobacteria* and *Actinobacteria* in non-responders was observed in this cohort. An outgrowth of these phyla has been associated with gastric bypass, metabolic disruption, inflammation, and cancer [21]. By contrast, the proportion of *Bacteroidetes* phylum decreased in non-responders compared with responders. An unexpected result was the detection of an elevation of *Firmicutes* phylum biodiversity in non-responders compared with responders. This finding conflicts with several studies that demonstrated a reduced concentration of the *Firmicutes* phylum in non-responders [18].

At the family level, the non-responders showed a remarkable decline in the *Bacteroidaceae* and *Lactobacillaceae* populations. Members of the *Lactobacillaceae* family have an essential role in the fermentation of kefir, which has been suggested to be a probiotic that may play a role in the treatment of IBD [22]. However, an increased abundance of *Bifidobacteriaceae*, *Lachnospiraceae*, *Enterobacteriaceae*, and *Muribaculaceae* was observed in non-responders compared to responders. A logical interpretation of the presence of increased populations of SCFA-producing bacteria including *Bifidobacteriaceae*, *Lachnospiraceae*, and *Muribaculaceae* in primary non-responders is a shift toward harboring pro-inflammatory bacteria, thereby promoting inflammation and ultimately leading to worsening conditions in primary non-responders [19].

A variation in the diversity of genera within one family – the *Lachnospiraceae* family – was detected where intestinal genera had a varied presence in non-responders. The abundance of *Roseburia* and *Coprococcus* genera decreased while the concentration of *Blautia* and *Clostridium* increased. Furthermore, a variation in abundance was observed in the Ruminococcaceae family: Where the diversity of *Feaclbacterium* genus increased while the diversity of the *Ruminococcus* genus decreased. This is consistent with some previous studies [23].

The *Enterococcus* genus is considered an aggressive genus that is characteristically highly abundant in the fecal samples of non-responders. The virulence and bacteriocinogenic features of the *Enterococcus* genus have been detected in the human gut. The *Enterococcus* genus has been associated with intestinal inflammation and a number of other infections, such as bloodstream and urinary tract infections, endocarditis, and peritonitis [24]. The decreased abundance of *Escherichia* was observed in primary non-responders compared with responders in the present study, which is inconsistent with several previous IBD studies [25]. Aside from these findings, the *Fusobacterium* genus increased in non-responsive patients, which coheres with some previous studies [26].

The concentration of *Klebsiella* and *Proteus* increased in primary non-responders. However, the *Bacteroides fragilis* (spp.) population, a human commensal bacterial, decreased in non-responders. This result corresponds with the previous studies [27]. A deficiency in several SCFA-producing bacteria in the fecal samples of non-responders was found, including the genera *Anaerostipes*, *Coprococcus*, *Lachnospira*, *Roseburia*, and *Sutterella*. This outcome is in line with a number of the previous studies’ [28]. SCFA-producing bacteria contribute to shaping the architecture of the gut community, organize transepithelial transport, are important for intestinal motility, and regulate the microbial homeostasis within the intestine. Moreover, SCFA has important immunomodulatory and anti-inflammatory characteristics [29]. Despite the fact that *B. animalis* and *Faecalibacterium prausnitzii* are used as probiotics, an increase in the concentration of these species was observed in primary non-responders and therefore may be used as a biomarker (*B. animalis*) for similar patients. This increase may be interpreted in rare cases as a result of high consumption of probiotics, associated with a high risk of adverse reactions between microbiota and their hosts. Consequently, this phenomenon may be linked with a number of diseases, such as bacterial infections and sepsis [30].

The *Desulfovibrio* genus, which belongs to the Proteobacteria phylum, is heterogeneous sulfate-reducing bacteria [31]. An increase in the *Desulfovibrio* genus has been associated with an elevated glutathione and riboflavin metabolism [32], rising toxins production, and increased bacterial genes attached with virulence agents. In the present study, the abundance of the *Desulfovibrio* genus was increased in non-responders compared with responders. The high diversity of *Desulfovibrio* in such patients is consistent with a number of other studies [33].

The present study is limited by its small sample size and lack of TDM measurements, which is typically used to document adequate trough levels and absence of anti-drug antibodies that are necessary to prove primary nonresponse. Nevertheless, this is the first study to examine the microbiota of Saudi IBD patients as potential biomarkers for response to anti-TNF therapy and as such can help pave the way for further larger studies.

## CONCLUSION

The fecal microbiota of primary non-responders to anti-TNF-α therapy demonstrated dysbiosis, in addition to other features, such as decreased biodiversity, augmentation in opportunistic pathogenic microbiota, and a lack of SCFA-producing bacteria. A decreased abundance of SCFA-producing bacteria, including *Anaerostipes*, *Coprococcus*, *Lachnospira*, *Roseburia*, and *Ruminococcus*, was detected in unresponsive patients. Particular fecal microbiota may be used as biomarkers to predict clinical response to anti-TNF-α medication. The potential biomarkers for primary non-response patients identified were *Klebsiella*, *Eubacteriaceae*, *RF32, B. animalis*, and *Muribaculaceae*.

## Figures and Tables

**TABLE S1:**
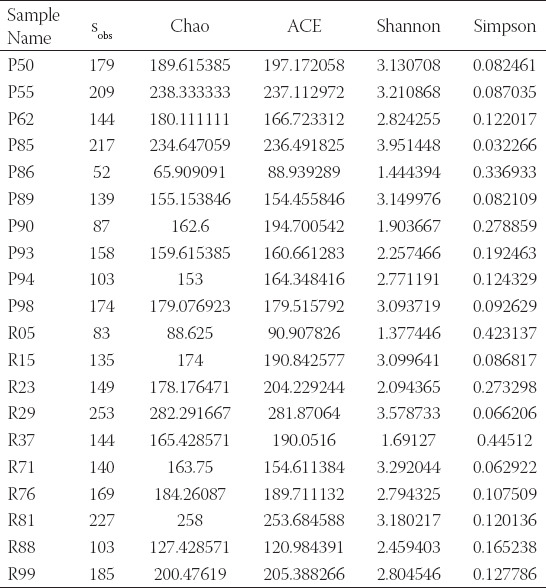
Alpha-diversity of the microbial communities’ composition among 20 stool samples of IBD patients.

**TABLE S2:**
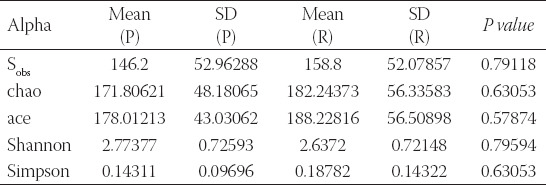
Statistical analysis of alpha diversity comparison results among 20 faecal samples of IBD patients.
